# Use of a new non-contrast-enhanced BOOST cardiac MR sequence before electrical cardioversion or ablation of atrial fibrillation—a pilot study

**DOI:** 10.3389/fcvm.2023.1177347

**Published:** 2023-06-16

**Authors:** Gábor Orbán, Zsófia Dohy, Ferenc Imre Suhai, Anikó Ilona Nagy, Zoltán Salló, Márton Boga, Máté Kiss, Karl Kunze, Radhouene Neji, Rene Botnar, Claudia Prieto, László Gellér, Béla Merkely, Hajnalka Vágó, Nándor Szegedi

**Affiliations:** ^1^ Heart and Vascular Center, Semmelweis University, Budapest, Hungary; ^2^ Siemens Healthcare Hungary, Budapest, Hungary; ^3^ MR Research Collaborations, Siemens Healthcare Limited, Frimley, United Kingdom; ^4^ School of Biomedical Engineering and Imaging Sciences, King’s College London, London, United Kingdom; ^5^ Escuela de Ingeniería, Pontificia Universidad Católica de Chile, Santiago, Chile

**Keywords:** atrial fibrillation, ablation, pulmonary vein anatomy, left atrial thrombus, cardiac magnetic resonance imaging, BOOST sequence

## Abstract

**Introduction:**

Left atrial appendage (LAA) thrombus is the most common source of embolization in atrial fibrillation (AF). Transesophageal echocardiography (TEE) is the gold standard method for LAA thrombus exclusion. Our pilot study aimed to compare the efficacy of a new non-contrast-enhanced cardiac magnetic resonance (CMR) sequence (BOOST) with TEE for the detection of LAA thrombus and to evaluate the usefulness of BOOST images for planning radiofrequency catheter ablation (RFCA) compared with left atrial (LA) contrast-enhanced computed tomography (CT). We also attempted to assess the patients' subjective experiences with TEE and CMR.

**Methods:**

Patients with AF undergoing either electrical cardioversion or RFCA were enrolled. Participants underwent pre-procedural TEE and CMR scans to evaluate LAA thrombus status and pulmonary vein anatomy. Patient experiences with TEE and CMR were assessed using a questionnaire developed by our team. Some patients scheduled for RFCA also had pre-procedural LA contrast-enhanced CT. In such cases, the operating physician was asked to subjectively define the quality of the CT and CMR scan on a scale of 1–10 (1 = worst, 10 = best) and comment on CMR's usefulness in RFCA planning.

**Results:**

Seventy-one patients were enrolled. In 94.4%, both TEE and CMR excluded, and in 1 patient, both modalities reported the presence of LAA thrombus. In 1 patient, TEE was inconclusive, but CMR excluded LAA thrombus. In 2 patients, CMR could not exclude the presence of thrombus, but in 1 of those cases, TEE was also indecisive. During TEE, 67%, during CMR, only 1.9% of patients reported pain (*p* < 0.0001), and 89% would prefer CMR in case of a repeat examination. The quality of the left atrial contrast-enhanced CT scans was better compared with the image quality of the CMR BOOST sequence [8 (7–9) vs. 6 (5–7), *p* < 0.0001]. Still, the CMR images were useful for procedural planning in 91% of cases.

**Conclusion:**

The new CMR BOOST sequence provides appropriate image quality for ablation planning. The sequence might be useful for excluding larger LAA thrombi; however, its accuracy in detecting smaller thrombi is limited. Most patients preferred CMR over TEE in this indication.

## 1. Introduction

Atrial fibrillation (AF) is adults' most common sustained cardiac arrhythmia worldwide (1). In addition to its high incidence and mortality rate, its clinical significance stems from its severe complications (2–5). One of the most dreaded complications of AF is arterial embolization, which most often manifests as an ischemic stroke (6, 7). It is well known that the most common source of thromboembolism is a left atrial appendage (LAA) thrombus (8–10). An integral part of the management of AF is rhythm control, with non-pharmacological options such as electrical cardioversion (ECV) and radiofrequency catheter ablation (RFCA) (1). In the context of interventions, careful pre-procedural exclusion of LAA thrombus is essential to avoid peri- and post-procedural neurological complications (e.g., stroke) (11–13). Currently, transesophageal echocardiography (TEE) is the gold standard method for detecting LAA thrombus (14, 15). However, TEE is an invasive procedure requiring esophageal intubation, usually performed under conscious sedation. It may result in esophageal injury, in addition to the potential distress caused to the patient (16). Alternative techniques exist to replace TEE, such as intracardiac echocardiography (ICE), contrast-enhanced computed tomography (CT), and contrast-enhanced cardiac magnetic resonance (CMR) (17–19). Nevertheless, these imaging modalities also have significant drawbacks (20). Recently, a new CMR sequence, called Bright-blood and black-BlOOd phase SensiTive inversion recovery (BOOST) sequence, has been developed that does not require a contrast agent but still provides good image quality (21). Based on initial studies, it seems suitable for assessing both pulmonary vein (PV) anatomy (22), which feature may prove beneficial in the planning of RFCA procedures (23, 24), and detecting LAA thrombus (25). However, it has not yet been compared with the gold standard TEE examination in terms of LAA thrombus detection.

Our study aimed to compare the efficacy of CMR and TEE examinations for the detection of LAA thrombus and to assess the usefulness of CMR images for planning catheter ablation procedures compared with left atrial contrast-enhanced CT. In addition, an attempt was made to compare patients' subjective experiences with TEE and CMR (TEE and CMR examination distress questionnaire).

## 2. Methods

### 2.1. Patient population

Patients undergoing either ECV or RFCA due to symptomatic AF who had already been scheduled for TEE after outpatient assessment were enrolled in our single-center prospective study at the Heart and Vascular Center of Semmelweis University, Budapest, Hungary, between May 2021 and June 2022. Patients with any contraindications to CMR examination were excluded; thus, those with claustrophobia or magnetic foreign bodies, e.g., an implantable cardiac electronic device or an insulin pump, were excluded from the study. All enrolled patients underwent pre-procedural imaging, which included both TEE and CMR scans on the same day. In all cases, CMR was performed first, followed by TEE within 2–3 h. Both modalities were used to confirm or exclude the presence of an LAA thrombus and to compare the two imaging modalities. The CMR was also used to assess left atrial (LA) and PV anatomy. We also asked patients to complete a second questionnaire within one week of the imaging scans to evaluate their distress, pain, discomfort, and preference for TEE or CMR. Of note, patients scheduled for RFCA might have had a pre-procedural LA contrast-enhanced CT, depending on the operating physician's decision. In such cases, the operating physician was asked to subjectively define the quality of the CT and CMR scan of the given patient on a scale of 1–10, with 1 being the worst and 10 being the best rating. Moreover, their opinion was also asked on whether CMR is appropriate for procedural planning.

All patients agreed to the pre-procedural imaging and provided written consent to data retrieval and analysis. Ethics approval was obtained from the Hungarian Medical Research Council (No.: IV/4962-3/2021/EKU) and was in accordance with the Declaration of Helsinki.

### 2.2. TEE protocol and image analysis

TEE was performed with the patient in conscious sedation using an Epiq CV-X system equipped with either an X7-2T or an X8-2T xMATRIX transducer (8.0–2.0 MHz) positioned at the appropriate level within the esophagus. Images were recorded and stored on a PACS system for later evaluation. Multiple standard tomographic planes, including 0, 45, 90, and 135 degrees LAA focused views (and additional planes where appropriate), were imaged, paying meticulous attention to visualize all lobes. X-plane, 2D, and 3D B-mode images and color and spectral Doppler images were recorded. The color Doppler Nyquist limit was lowered according to the LAA flow velocity to enable visualization of flow within the LAA. The presence of LAA thrombus, spontaneous echo contrast (SEC), or sludge, as well as the LAA emptying velocity, was documented.

LAA thrombus was defined as a well-circumscribed solid echodensity, acoustically distinct from the underlying endocardium and pectinate muscles that were reproducibly visible from multiple imaging planes. By consensus, the presence of organized thrombus in the LAA tip could not be excluded in the presence of dense sludge. As the TEE is considered the gold-standard examination for excluding LAA thrombus, we used its result to validate the CMR.

### 2.3. CMR protocol and image analysis

CMR examinations were performed on a 1.5T MR scanner (MAGNETOM Aera, Siemens Healthcare, Germany). After semiautomated angulation, retrospectively gated balanced steady-state free precession (bSSFP) cine images were acquired in 2-chamber, 4-chamber, and left ventricle outflow tract views, additionally in a modified 2-chamber long axis slice optimized for the LAA. An ECG-triggered 3D whole-heart bright-blood and black-blood bSSFP (BOOST) prototype sequence was performed both with T2-preparation pre-pulse (T2prep) and MT-preparation (MTC) as previously described (21, 22). Image navigation (iNAV) was used for respiratory motion estimation and compensation. The BOOST measurements were performed with the coverage of the LA, including the ostia of PVs and the appendage at an isotropic resolution of 1.4 × 1.4 × 1.4 mm. No contrast agent was administered. In the case of artifacts unrelated to sustained patient conditions, the BOOST image acquisition was repeated. However, repeat imaging was not performed for motion artifacts in those cases where artifacts were caused by a sustained patient condition, e.g., persistent AF during CMR imaging.

CMR data were analyzed using Medis Suite 4.0 software (Medis Medical Imaging Software, Leiden, The Netherlands). On two- and four-chamber long-axis cine images, LA endocardial contour was traced manually, and the maximal and minimal LA volumes (maximum LAV, minimum LAV), LA stroke volume (LA SV), and LA ejection fraction (LAEF) were quantified. The LA volumes were standardized to the body surface area, yielding maximum body surface area-indexed LA volume (LAVi), minimum LAVi, and body surface area-indexed LA stroke volume (LA SVi). Using the 3D view application of the Medis Suite, the bidirectional diameters of pulmonary venous ostia were measured on the MTC BOOST bright-blood images, after which PV ostial areas were calculated. PV anatomy variations were defined as described previously (23). On T2prep images, the LAA was systematically inspected for possible thrombus. LAA thrombus was defined as a circumscribed lower signal intensity on the bright-blood images and a circumscribed higher signal intensity on the dark-blood images in the same localization.

The CMR image was also used to plan the ablation procedure in the RFCA patient group.

### 2.4. LA contrast-enhanced CT protocol

Some patients scheduled for RFCA also underwent LA contrast-enhanced CT to assess LA and PV anatomy at the discretion of the operating physician. In these cases, CT scans were usually performed before the CMR scans. LA contrast-enhanced CT examinations were performed with a 256-slice scanner (Brilliance iCT 256, Philips Healthcare, Best, The Netherlands) with prospective ECG-triggered axial acquisition mode. For cardiac CT, 100–120 kV with 200–300 mAs tube current was used based on the patient's body mass index. Image acquisition was performed with 128 × 0.625 mm detector collimation and 270 ms gantry rotation time. If necessary, oral or intravenous beta-blocker was administered for heart rate control before the CT scans. Mid-diastolic triggering was applied with 3%–5% padding (73%–83% of the R-R interval) in all included patients. Intravenous contrast agent (85–95 ml Iomeron 400, Bracco Ltd, Milan, Italy) at a flow rate of 4.5–5.5 ml/s from antecubital vein access via an 18-gauge catheter using a four-phasic protocol. Bolus tracking in the LA was used to obtain proper scan timing. CT data sets were reconstructed with 0.8 mm slice thickness with 0.4 mm increments. PV ostial areas were measured as described previously (23). For patients who underwent both CMR and contrast-enhanced CT, measurements of PV ostial areas were compared to assess the reliability of the values measured with BOOST.

### 2.5. TEE and CMR examination distress questionnaire

The TEE and CMR examination distress questionnaire was developed by our research team to assess and compare patients’ experiences in the two types of imaging examinations. The questionnaire consists of 20 questions divided into three domains. The first and second parts surveyed, among other things, the extent to which patients were informed about the examinations, the degree of mental and physical distress experienced during the imaging, and their impression of the length of the examinations. The same nine questions were asked regarding the TEE, then the CMR examination. Next, in the final domain, patients were also asked which imaging method they would prefer if they had to undergo one of them again. Then the last question with a free-text response inquired about the reason for their preference (for more details, see Supplementary Table S1).

The TEE and CMR examination distress questionnaire was completed within one week of the imaging studies. Interviews were conducted over the telephone, where the questions were explained to the patients by the clinical staff, and the staff also recorded their answers.

### 2.6. Data collection

In addition to demographic, anthropometric, and medical data, data from imaging studies and their analysis were also collected. Neurological complications [stroke or transient ischemic attack (TIA)] were also documented at the 3-month follow-up clinical visit.

### 2.7. Statistical analysis

Most of the variables showed non-parametric distributions after performing the Shapiro-Wilk test. Thus, the continuous variables were expressed as medians and interquartile ranges. Categorical variables are expressed as numbers and percentages. Fisher's exact test was performed to examine contingency between selected groups. Continuous variables were compared with the Mann-Whitney U test. A two-tailed *p*-value of <0.05 was considered statistically significant. Statistical analyses were performed using IBM SPSS 25 (Apache Software Foundation, USA) and GraphPad Prism 9.1.2 (GraphPad Softwares Inc., USA) software products.

## 3. Results

### 3.1. Characteristics of the study population

A total of 80 patients were screened, of which nine were excluded due to contraindications for CMR: five due to claustrophobia and four due to magnetic foreign bodies. Data from the 71 enrolled patients [age 65 (57–72) years, 38% female] were collected and analyzed. Fifty-two percent of patients had paroxysmal AF, the BMI was 28 (26–32) kg/m^2^, and 77% had hypertension. Two point eight percent of them had a prior stroke or TIA. Sixty-six percent of patients were admitted for RFCA and 34% for ECV. Baseline characteristics of the study population and the distribution of anticoagulants used before the examinations are shown in Table 1.

**Table 1:** Baseline characteristics of the study population.

Patient characteristics (*n* = 71)	
Age (years)	65 (57–72)
Female, *n* (%)	27 (38)
AF type	
Paroxysmal, *n* (%)	37 (52)
Persistent, *n* (%)	34 (48)
BMI (kg/m^2^)	28 (26–32)
Hypertension, *n* (%)	55 (77)
Hyperlipidemia, *n* (%)	36 (51)
Diabetes, *n* (%)	16 (23)
CAD, *n* (%)	15 (21)
Prior stroke/TIA, *n* (%)	2 (2.8)
Other thromboembolic events, *n* (%)	3 (4.2)
Thyroid gland disease, *n* (%)	13 (18)
Prior RFCA, *n* (%)	23 (32)
PVI, *n* (%)	16 (23)
CTI ablation, *n* (%)	3 (4.2)
PVI + CTI ablation, *n* (%)	4 (5.6)
GFR (ml/min/1.73 m^2^)	71 (60–89)
LVEF (%)	55 (52–59)
CHA_2_DS_2_-VASc score	2 (2–4)
Reason for admission	
RFCA, *n* (%)	47 (66)
ECV, *n* (%)	24 (34)
Anticoagulants used prior to imaging (*n* = 71)	
Rivaroxaban, *n* (%)	21 (29.6)
Apixaban, *n* (%)	20 (28.2)
Dabigatran, *n* (%)	12 (16.9)
Edoxaban, *n* (%)	9 (12.7)
Vitamin K antagonists, *n* (%)	5 (7)
LMWH, *n* (%)	1 (1.4)
None, *n* (%)	3 (4.2)

AF, atrial fibrillation; BMI, body mass index; CAD, coronary artery disease; TIA, transient ischemic attack; RFCA, radiofrequency catheter ablation; PVI, pulmonary vein isolation; CTI, cavotricuspid isthmus; GFR, glomerular filtration rate; LVEF, left ventricular ejection fraction; ECV, electrical cardioversion; LMWH, low-molecular-weight heparin. Continuous variables are expressed as medians and interquartile ranges.

### 3.2. Imaging results, detection of LAA thrombus by TEE and CMR

The whole CMR examination, consisting of localizers, cine images, T2prep, and MT BOOST sequences, took 40 (30–45) minutes, while the duration of the TEE examination (measured from the transducer's introduction until its removal) was 7 (5–9) min. Some or all parts of the BOOST image acquisition had to be repeated in 10 patients due to technical difficulties or artifacts that were not related to a sustained patient condition such as persistent AF, during CMR imaging.

**Figure 1:**
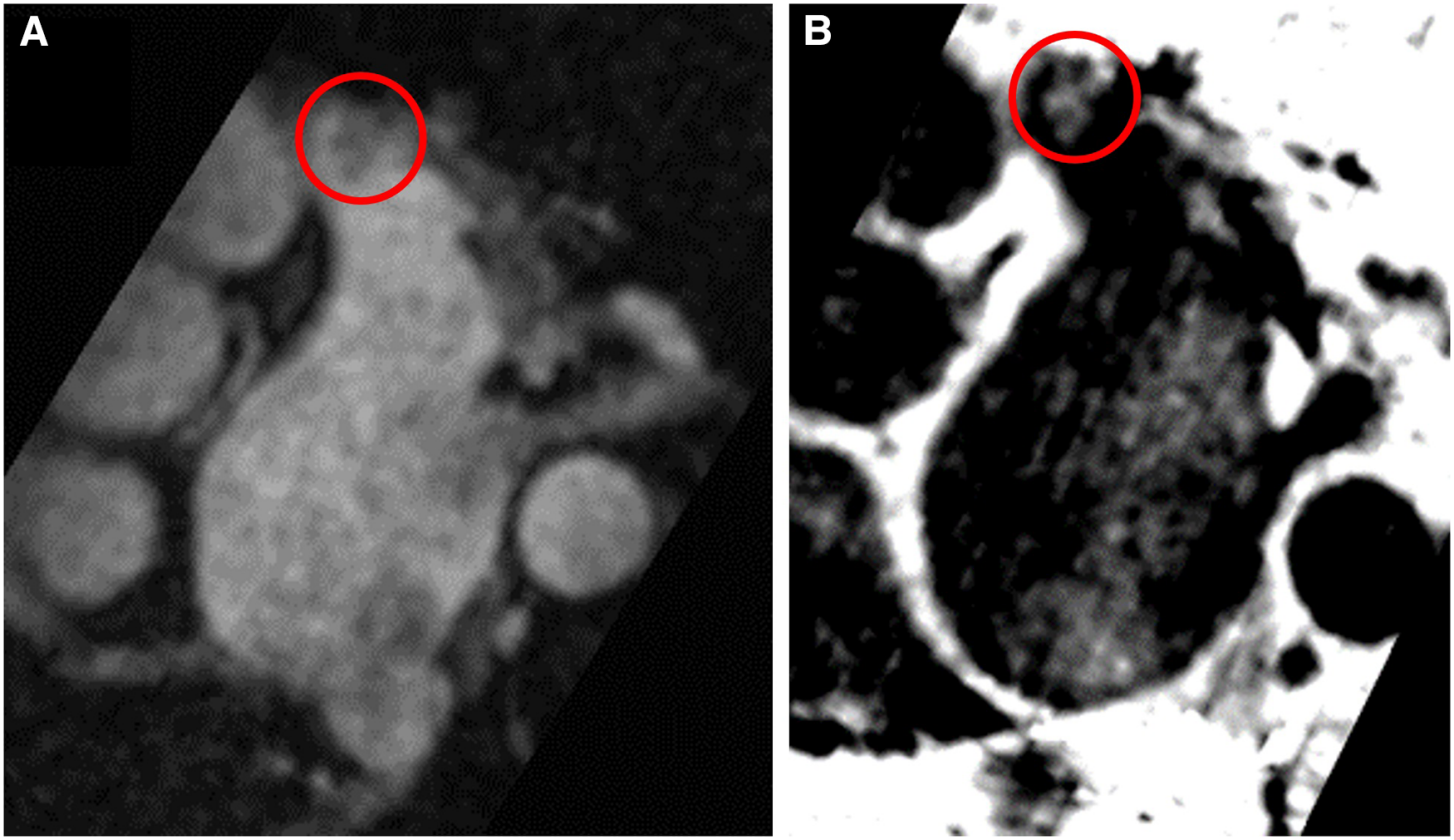
Left atrial appendage thrombus circled in red on CMR T2-preparation (T2prep) BOOST sequence images. Panel (**A**): Magnitude image of T2prep BOOST. Panel (**B**): Dark-blood image of T2prep BOOST (Heart and Vascular Center, Semmelweis University).

**Figure 2:**
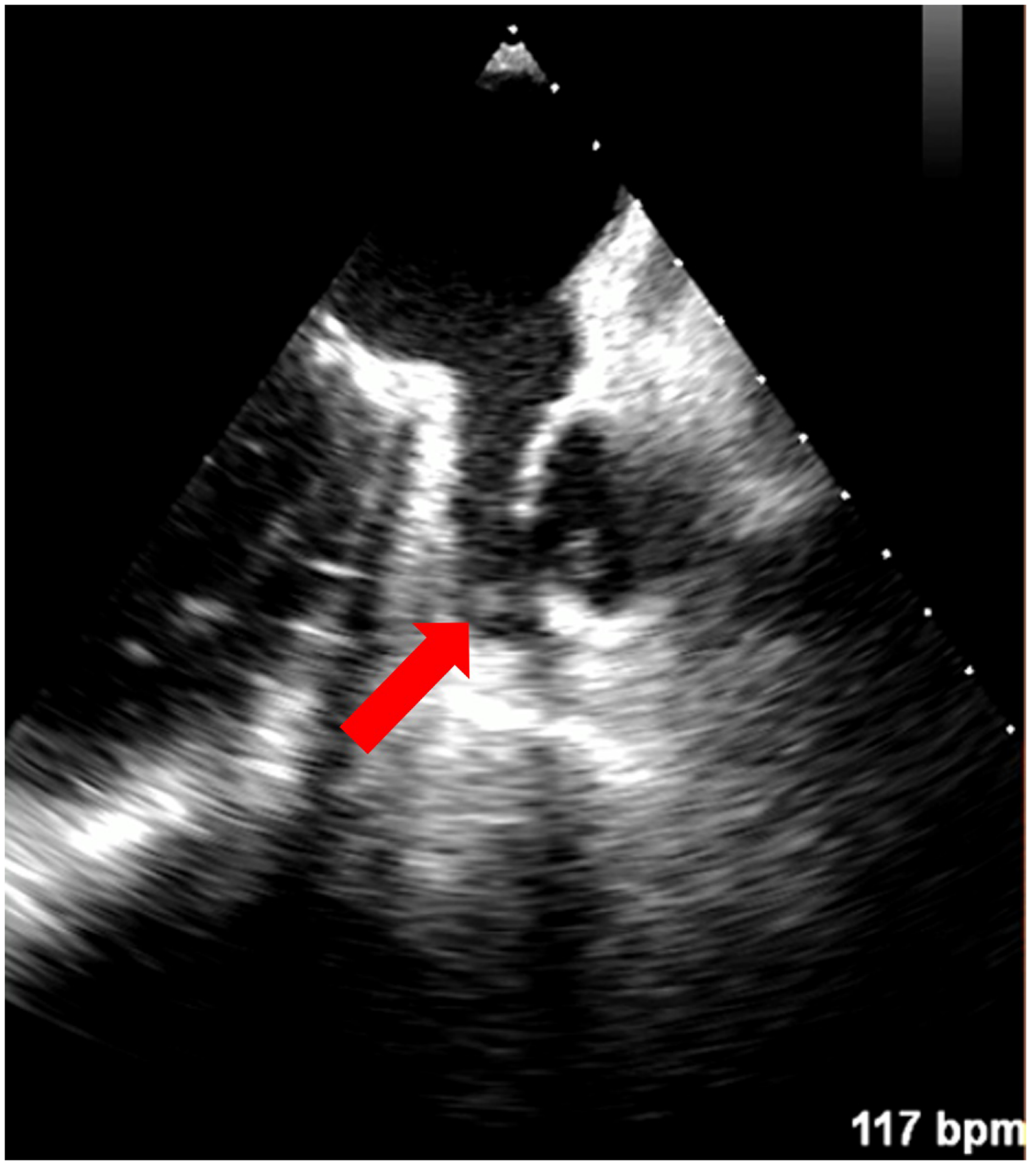
Left atrial appendage thrombus indicated with a red arrow on TEE (Heart and Vascular Center, Semmelweis University).

In 67 patients (94.4%), both TEE and CMR excluded the presence of LAA thrombus. In 1 patient (1.4%), both TEE and CMR showed the presence of thrombus [Figures 1, 2; a supplementary movie file shows the thrombus detected with TEE in motion (see Supplementary Video S1)].

In 1 (1.4%) patient, examined with all three imaging modalities (TEE, CMR, contrast-enhanced LA CT), TEE could not exclude LAA thrombus due to dense sludge. CT was also inconclusive, whereas the BOOST CMR sequence clearly excluded the presence of LAA thrombus. Three months later (without any changes in the patient's anticoagulant regime), repeated TEE and contrast-enhanced CT both described the absence of LAA thrombus, and ablation was successfully performed without thromboembolic complications.

In 2 patients (2.8%), CMR could not rule out the presence of a thrombus with certainty. In both cases, images of poor quality were obtained due to motion artifacts produced by the ongoing arrhythmia during CMR imaging. In one of these two patients, TEE was also indecisive due to dense sludge in the LAA. However, in the other patient, TEE clearly showed the presence of LAA thrombus.

No stroke or TIA occurred either peri- or post-procedurally during the three-month follow-up of patients. The imaging results are shown in Table 2.

**Table 2:** Imaging results.

Imaging results (*n* = 71)	
CMR duration (min)	40 (30–45)
TEE duration (min)	7 (5–9)
LAA emptying velocity (cm/sec)	53 (37–71)
SEC on TEE, *n*	4
Both TEE and CMR excluded thrombus, *n*	67
Both TEE and CMR showed thrombus, *n*	1
Inconclusive TEE result, but CMR excluded thrombus, *n*^a^	1
Both TEE and CMR were uncertain about thrombus, *n*^a^	1
Inconclusive CMR result, but TEE showed thrombus, *n*	1

^a^ In these cases, the presence of organized thrombus could not be excluded with TEE due to dense sludge.

CMR, cardiac magnetic resonance; TEE, transesophageal echocardiography; LAA, left atrial appendage; SEC, spontaneous echo contrast. Continuous variables are expressed as medians and interquartile ranges.

### 3.3. PV anatomy pattern

In our study group, 47 patients (66.2%) showed normal anatomy with four separate PV ostia. In 17 patients (23.9%), ostia of the two left PVs were merged to form a common trunk. This pattern was the most frequent variation; left short common trunk (LSCT) was seen in 14 (19.7%) and left long common trunk (LLCT) in 3 patients (4.2%). In one patient (1.4%), we found a right common trunk (RCT). Right accessory PVs were observed in 9 cases (12.7%). Overall, 3 patients (4.2%) presented with a combination of variant branching patterns, consisting of 1 patient (1.4%) with both LSCT and right middle pulmonary vein (RMPV), 1 patient (1.4%) with a concurrent LLCT and RMPV, and 1 patient (1.4%) with both LSCT and RCT (Table 3).

**Table 3:** Pulmonary vein anatomy variations.

PV anatomy variations (*n* = 71)	
Normal PV anatomy, *n* (%)	47 (66.2)
Variant PV anatomy, *n* (%)	24 (33.8)
Only LSCT, *n* (%)	12 (16.9)
Only LLCT, *n* (%)	2 (2.8)
Only RUPV, *n* (%)	1 (1.4)
Only RMPV, *n* (%)	6 (8.5)
LSCT + RMPV, *n* (%)	1 (1.4)
LSCT + RCT, *n* (%)	1 (1.4)
LLCT + RMPV, *n* (%)	1 (1.4)

PV, pulmonary vein; LSCT, left short common trunk; LLCT, left long common trunk; RUPV, right upper accessory PV; RMPV, right middle PV; RCT, right common trunk.

### 3.4. PV ostial areas and LA parameters

The areas of the PV ostia regarding all enrolled patients are detailed in Table 4.

**Table 4:** Pulmonary vein ostial areas of all enrolled patients measured with CMR.

PV ostial areas (mm^2^)	
RSPV	299 (262–386)
RIPV	284 (219–378)
LSPV	258 (201–346)
LIPV	253 (185–376)
LSCT	613 (471–697)
LLCT	584 (401–679)
RCT	280
RUPV	251
RMPV	77 (57–114)

CMR, cardiac magnetic resonance; PV, pulmonary vein; RSPV, right superior PV; RIPV, right inferior PV; LSPV, left superior PV; LIPV, left inferior PV; LSCT, left short common trunk; LLCT, left long common trunk; RCT, right common trunk; RUPV, right upper accessory PV; RMPV, right middle PV. Continuous variables are expressed as medians and interquartile ranges.

Twenty-five patients underwent both LA contrast-enhanced CT and CMR before ablation. In their cases, there was no significant difference between PV ostial areas measured on CT and BOOST CMR images (all *p* > 0.05) (Table 5).

**Table 5:** Differences between pulmonary vein ostial areas in cases where both CMR and contrast-enhanced CT were performed (*n* = 25).

	BOOST CMR	Contrast-enhanced CT	*p*-value
RSPV (mm^2^)	299 (267–393)	312 (269–377)	0.51
RIPV (mm^2^)	284 (244–394)	309 (244–439)	0.64
LSPV (mm^2^)	267 (208–321)	276 (208–350)	0.71
LIPV (mm^2^)	268 (213–392)	274 (247–393)	0.69

CMR, cardiac magnetic resonance; CT, computed tomography; BOOST, Bright-Blood and Black-Blood Phase Sensitive; RSPV, right superior pulmonary vein; RIPV, right inferior pulmonary vein; LSPV, left superior pulmonary vein; LIPV, left inferior pulmonary vein. Continuous variables are expressed as medians and interquartile ranges.

During CMR imaging, 37 patients (52%) were in sinus rhythm (SR), 2 patients (2.8%) were in atrial flutter, and 32 patients (45%) were in AF. LA ejection fraction (LAEF) was 53 (44–60) % in patients in sinus rhythm, while it was 19 (12–29) % in patients in non-SR. Minimum and maximum body surface area-indexed LA volume (LAVi) were 26 (21–51) ml/m^2^ and 50 (43–67) ml/m^2^, respectively. Body surface area-indexed LA stroke volume (LA SVi) was 17 (11–25) ml/m^2^. Details of the measurements are shown in Table 6.

**Table 6:** Left atrial parameters measured with CMR.

Left atrial parameters	
Patients in SR during CMR, *n* (%)	37 (52)
Patients in non-SR during CMR, *n* (%)	34 (48)
LAEF of patients in SR (%)	53 (44–60)
LAEF of patients in non-SR (%)	19 (12–29)
Minimum LAV (ml)	53 (43–107)
Maximum LAV (ml)	106 (87–137)
Minimum LAVi (ml/m^2^)	26 (21–51)
Maximum LAVi (ml/m^2^)	50 (43–67)
LA SV (ml)	35 (26–52)
LA SVi (ml/m^2^)	17 (11–25)

CMR, cardiac magnetic resonance; SR, sinus rhythm; LAEF, left atrial ejection fraction; LAV, left atrial volume; LAVi, body surface area-indexed left atrial volume; LA SV, left atrial stroke volume; LA SVi, body surface area-indexed left atrial stroke volume. Continuous variables are expressed as medians and interquartile ranges.

### 3.5. Electrophysiologists' evaluation of the usefulness of LA contrast-enhanced CT vs. CMR regarding RFCA planning

**Figure 3:**
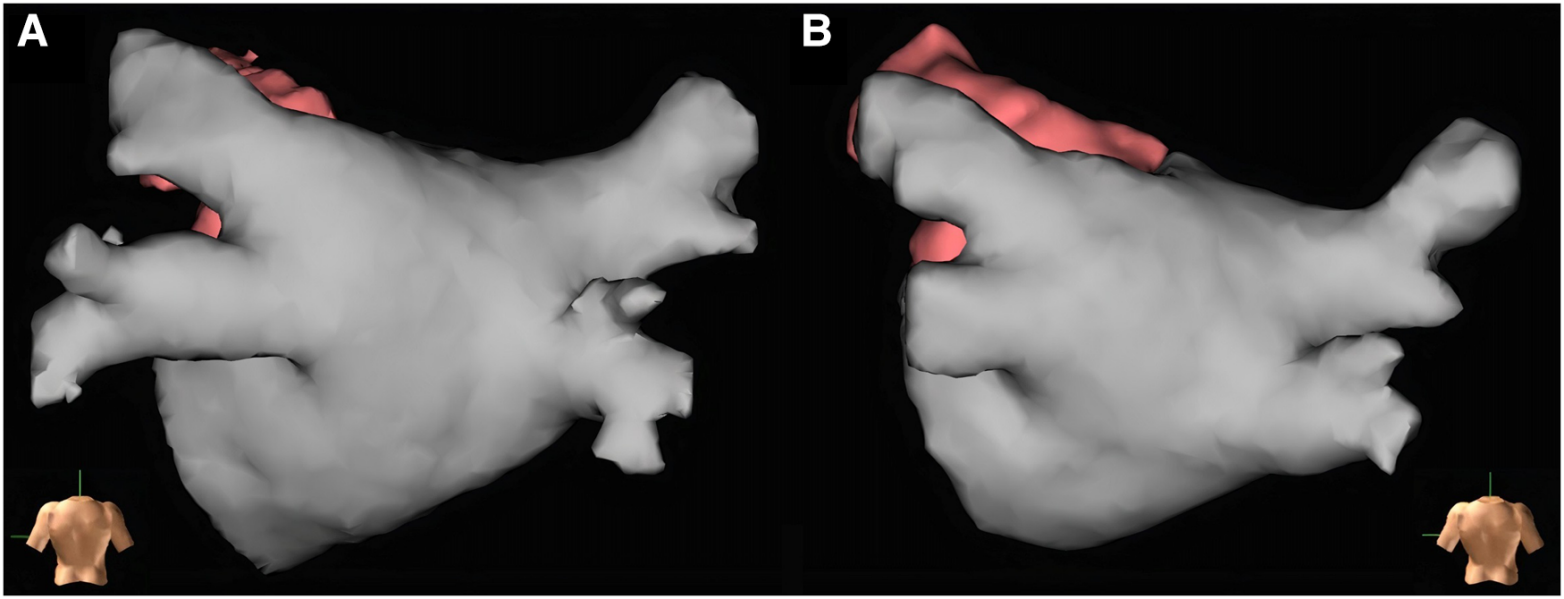
Left atrial reconstructions before radiofrequency catheter ablation for procedural planning based on left atrial contrast-enhanced CT and CMR BOOST bright-blood images created with EnSite Precision electroanatomical mapping system. Both reconstructions show the left atrium of the same patient (Heart and Vascular Center, Semmelweis University). Panel (**A**): Reconstruction based on left atrial contrast-enhanced CT images. Panel (**B**): Reconstruction based on left atrial CMR BOOST bright-blood images.

Twenty-five patients had both LA contrast-enhanced CT and CMR before the ablation procedure. Based on the operating electrophysiologists' subjective opinion (1–10 scale), the quality of the LA contrast-enhanced CT scans was better compared with the image quality of the non-contrast-enhanced CMR BOOST sequence [8 (7–9) vs. 6 (5–7), *p* < 0.0001] (Figures 3A, B**)**. Still, they found the CMR images appropriate for procedural planning in 43 out of 47 cases (91%).

### 3.6. Results of the TEE and CMR examination distress questionnaire

Fifty-four patients (76%) completed the TEE and CMR examination distress questionnaire. Thirty-six patients (67%) had at least mild pain during the TEE, while only one patient (1.9%) experienced mild discomfort during CMR (*p* < 0.0001). Half of the patients felt reluctant to repeat the TEE, whereas this number was 7.4% in the case of CMR (*p* < 0.0001). Most of the patients (89%) reported that they would choose CMR over TEE if they had to undergo the examination once again. Those who preferred CMR found it more comfortable, not associated with unpleasant physical impact and did not cause gagging and nausea. Those who opted for TEE argued that the CMR scan was scary or took much longer. Detailed responses of the patients are provided in Supplementary Table S2.

## 4. Discussion

### 4.1. Main findings

Our experimental study is the first to investigate the new CMR BOOST sequence in a large number of AF patients. We found the sequence suitable for assessing LA and PV anatomy and, thus, for ablation planning; the image quality was appropriate in 91% of the cases. Furthermore, the sequence might also be suitable for excluding larger LAA thrombi.

### 4.2. LAA thrombus assessment

RFCA and ECV are fundamental elements in the complex management of AF as non-pharmacological rhythm control interventions. In both cases, pre-procedural imaging is crucial to exclude the presence of LAA thrombus (1). To date, TEE has been considered the gold standard imaging modality to exclude LAA thrombus with a 97% sensitivity and 100% specificity (14, 15). In our clinic, a TEE examination is performed on the day of the ECV or RFCA procedure, and it is highly effective in preventing thromboembolic complications. However, TEE requires esophageal intubation; moreover, it is associated with non-negligible distress to the patient and can be complicated by esophageal injury (16). Although there are alternatives to TEE, such as ICE and contrast-enhanced CT or CMR, significant drawbacks of the latter were reported (17, 19, 26, 27). ICE is an invasive and expensive procedure, most commonly used to assist in performing a transseptal puncture for left atrial RFCA (28, 29). The ability of contrast-enhanced CT to detect LAA thrombus has been confirmed by several studies and meta-analyses (26, 27, 30). On the other hand, it is associated with radiation exposure, contrast allergy, and the worsening of chronic kidney disease that is often associated with atrial fibrillation (31). Similarly, contrast-enhanced CMR (especially delayed-enhancement CMR) has been shown to be effective in detecting LAA thrombus. However, its use is also limited by renal function and rarely by allergic reactions (19, 32).

On the other hand, the new non-contrast-enhanced CMR sequence (BOOST), which has been recently developed and has shown promising preliminary results, might help us to overcome these limitations (21, 22). As CMR does not use ionizing radiation, it is preferable for patients of fertile age, especially women. Moreover, this sequence does not use a contrast agent; thus, it is not limited by renal function or allergic reactions, and it provides two image series (bright- and black-blood series) within one acquisition.

Based on our current study, the new BOOST sequence may be able to exclude LAA thrombus: in 94.4% of patients, it was able to exclude the presence of thrombus in line with TEE, confirming BOOST's thrombus exclusion ability (Table 2).

This study is the initial evaluation of BOOST, and the validation technique is TEE. Thus, in the case of the one patient with TEE being inconclusive and BOOST excluding LAA thrombus, we cannot directly assume that the patient did not have an LAA thrombus just based on the absence of clinically manifest thromboembolic event during a three-month period. As contrast-enhanced CT was also inconclusive in this case, the use of further imaging (delayed-enhancement CMR) could have been an option to verify the LAA's thrombus-free status.

**Figure 4:**
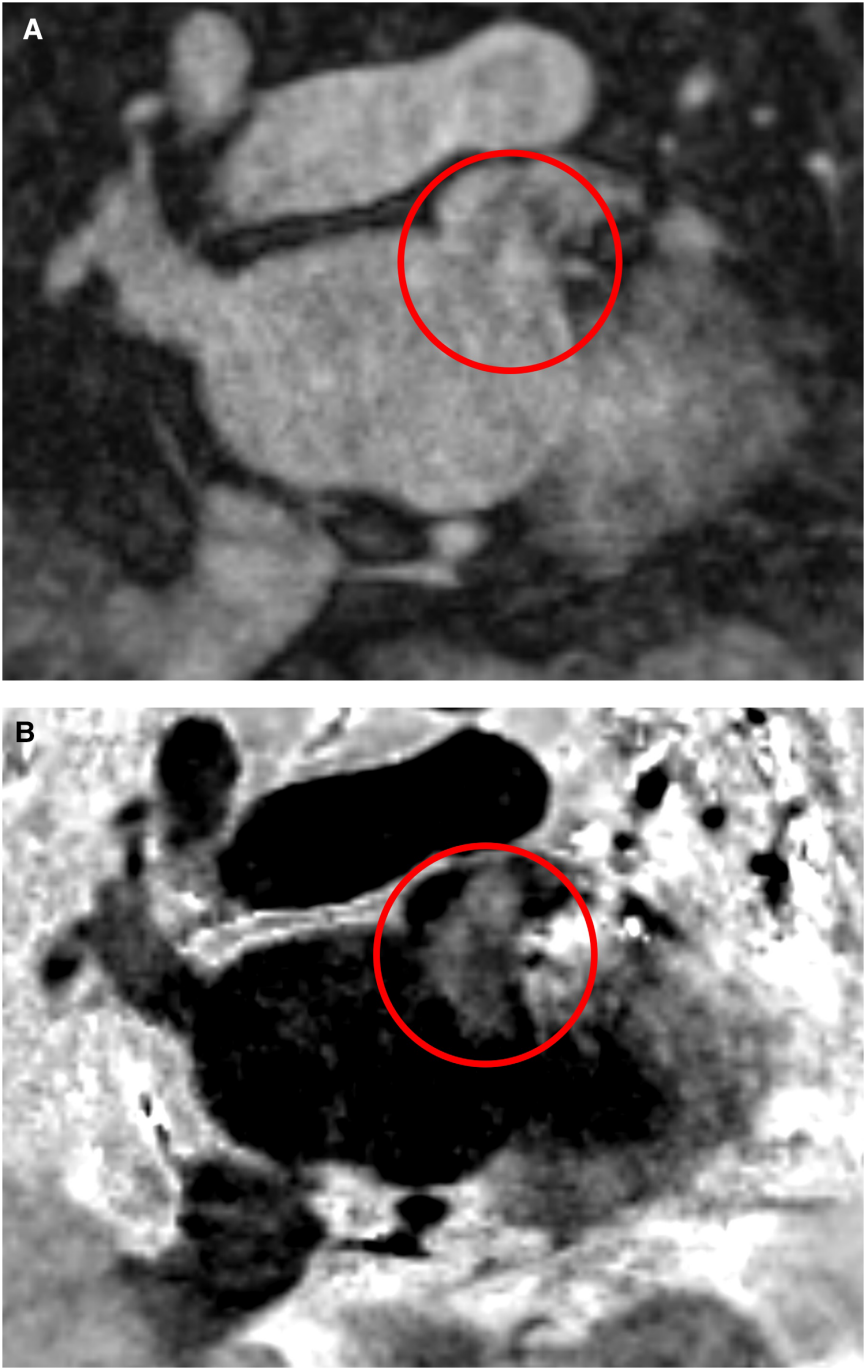
Left atrial appendage thrombus circled in red on CMR MT-preparation (MTC) BOOST sequence images. Panel (**A**): Bright-blood image of MTC BOOST. Panel (**B**): Dark-blood image of MTC BOOST (Heart and Vascular Center, Semmelweis University).

In summary, the new BOOST sequence is suitable for detecting larger thrombi, but its effectiveness in detecting or excluding smaller thrombi may be limited with the used spatial resolution settings. As we used an isotropic resolution of 1.4 × 1.4 × 1.4 mm in BOOST measurements, it is possible that the use of a higher resolution (1.2 × 1.2 × 1.2 mm) could improve its effectiveness in detecting smaller thrombi. Thus, further studies will be needed to characterize the sequence more accurately in LAA thrombus detection. The ability to detect larger thrombi is also supported by the case of a patient who was examined with the BOOST CMR sequence outside the scope of this study (Figure 4). Although the BOOST image was performed with MT-preparation (MTC), which is not the most suitable for thrombus detection, the large thrombus located in the left atrium is clearly visible.

In addition, when choosing the appropriate imaging modality (e.g., TEE or BOOST) for LAA assessment, comorbidities and potential contraindications should always be considered. Moreover, longer imaging time associated with BOOST CMR and overall accessibility of CMR should also be considered when selecting the imaging modality (Table 2).

### 4.3. PV anatomy assessment with the BOOST sequence prior to RFCA

In addition to ruling out LAA thrombus, imaging prior to RFCA has an essential role in procedural planning by assessing LA anatomy. Certain atrial and PV variations have been shown to influence the recurrence of AF after ablation (23, 24, 33–35). In addition, PV variations may also influence the choice of ablation tools (e.g., the feasibility of cryoballoon ablation, 31- or 35-mm device for FARAPULSE pulsed field ablation) and ablation outcomes. Thus, pre-procedural imaging to assess LA anatomy before AF ablation is common practice (13, 36, 37). Currently, the standard imaging examination used for AF ablation planning at our clinic is contrast-enhanced LA CT. We interviewed cardiac electrophysiologists at our clinic who used both contrast-enhanced LA CT and non-contrast-enhanced CMR BOOST sequences for pre-ablation imaging. They reported that, although image quality was inferior to contrast-enhanced CT, the CMR BOOST sequence imaging still provided adequate information on LA and PV anatomy for ablation planning (Figures 3A, B). The reliability of BOOST in RFCA planning and measurements is also supported by the fact that no significant difference was found in PV ostial area assessment compared with contrast-enhanced CT (all *p* > 0.05) (Table 5). Thus, in the future, the BOOST sequence may be an alternative to TEE for excluding LAA thrombi and might also replace contrast-enhanced CT for determining LA anatomy before catheter ablation. This might be especially true in young patients with a greater concern regarding ionizing radiation and in cases of impaired renal function.

### 4.4. Level of distress and discomfort associated with imaging, patient preference

As previously stated, TEE requires esophageal intubation. Although local anesthetics and anxiolytics are used to reduce discomfort associated with the examination, patients might suffer considerable discomfort and pain. Our results indicated that patients were significantly more likely to experience pain during TEE than during CMR (*p* < 0.0001). We also showed that the majority of patients (89%) would prefer to undergo CMR if given a choice due to less discomfort, pain, and lack of physical intrusion (see Supplementary Table S2). Thus, the CMR BOOST sequence may, in the future, be a favorable alternative to TEE, not only because of its accuracy but also considering the patients' choices. Of note, this has to be validated in a larger patient population.

### 4.5. Limitations

There are limitations to our work. It was a single-center observational study with a relatively low number of patients. Due to the low rate of LAA thrombi, the positive predictive value of the CMR BOOST sequence could not be adequately evaluated. Thus, further investigation of our findings on a large patient cohort is warranted. It is important to investigate the ability of BOOST with higher spatial resolution in the detection of smaller thrombi. The follow-up period was relatively short (3 months); however, sufficient to assess the procedure-related (RFCA or ECV) neurological events.

## 5. Conclusion

This is the first pilot study to investigate the new CMR BOOST sequence with respect to its thrombus detection ability, compared with TEE, and its usefulness in the pre-procedural planning of AF ablation procedures. We showed that the BOOST sequence provides appropriate image quality for RFCA planning. In addition, the sequence proves to be suitable for excluding larger LAA thrombi. However, its effectiveness in detecting smaller thrombi may be limited, and further studies are warranted. Moreover, based on the distress questionnaire, the majority of patients would prefer CMR over TEE.

## Data Availability

The raw data supporting the conclusions of this article will be made available by the authors, without undue reservation.
