# Supplementary material for: Use of a new non-contrast-enhanced BOOST cardiac MR sequence before electrical cardioversion or ablation of atrial fibrillation—a pilot study
Source: Front Cardiovasc Med. 2023 Jun 16;10:1177347. doi: 10.3389/fcvm.2023.1177347 (PMC10311645; doi:10.3389/fcvm.2023.1177347)
Supplement: Supplementary file 1 [file Table1.docx]

**Supplementary file 1**

**TEE and CMR examination distress questionnaire description**

**A, TEE:**

**First, please answer the questions regarding the transesophageal echocardiography:**

**Question 1**: How informed were you about the reason(s) for the examination?

1 - none

2 - to some extent, but overall, the reason was not fully clear

3 - to a moderate degree, I understood the reason, but I was left with many questions

4 - to a fair extent, but some questions remained unanswered

5 - to a full extent and all my questions were answered

**Question 2**: How informed were you about the examination and risks of it?

1 - not at all

2 - to some extent, but overall, not fully clear

3 - to a moderate degree, I understood them, but I still had many questions

4 - to a fair extent, but some questions remained unanswered

5 - to a full extent and all my questions were answered

**Question 3**: To what extent were you afraid of the investigation based on the information you had heard beforehand?

1 - I was not afraid at all

2 - I was slightly afraid

3 - I was moderately afraid

4 - I was definitely afraid

5 - I was terrified of the examination

**Question 4**: Were you given any sedative/anxiolytic medication before the the transesophageal echocardiography examination?

0 - yes

1 - no

**Question 5**: How well did the examination match what you heard in the preliminary information?

1 - not at all

2 - slightly matched

3 - moderately matched

4 - mostly matched

5 - completely matched

**Question 6**: How painful was the examination?

1 - no pain

2 - a little pain

3 - moderately painful

4 - very painful, difficult to tolerate

5 - almost unbearable

**Question 7**: How anxious/afraid were you during the examination?

1 - no anxiety/fear at all

2 - I was slightly anxious/scared

3 - I was moderately anxious/scared

4 - I was definitely anxious/scared

5 - terribly anxious/scared: couldn't wait for it to be over

**Question 8**: How long did you feel the examination was?

1 - it was not long

2 - it was of reasonable length

3 - it was long

**Question 9**: If you had to go through the examination again, how afraid would you be?

1 - I would not be afraid at all

2 - I would be slightly afraid

3 - I would be moderately afraid

4 - I would definitely be afraid

5 - I would be terrified

**B, CMR:**

**Please also answer the following questions about the cardiac MR scan:**

**Question 10**: How informed were you about the reason(s) for the examination?

1 - none

2 - to some extent, but overall, the reason was not fully clear

3 - to a moderate degree, I understood the reason, but I was left with many questions

4 - to a fair extent, but some questions remained unanswered

5 - to a full extent and all my questions were answered

**Question 11**: How informed were you about the examination and risks of it?

1 - not at all

2 - to some extent, but overall, not fully clear

3 - to a moderate degree, I understood them, but I still had many questions

4 - to a fair extent, but some questions remained unanswered

5 - to a full extent and all my questions were answered

**Question 12**: To what extent were you afraid of the investigation based on the information you had heard beforehand?

1 - I was not afraid at all

2 - I was slightly afraid

3 - I was moderately afraid

4 - I was definitely afraid

5 - I was terrified of the examination

**Question 13**: Were you given any sedative/anxiolytic medication before the cardiac MRI examination?

0 - yes

1 - no

**Question 14**: How well did the examination match what you heard in the preliminary information?

1 - not at all

2 - slightly matched

3 - moderately matched

4 - mostly matched

5 - completely matched

**Question 15**: How painful was the examination?

1 - no pain

2 - a little pain

3 - moderately painful

4 - very painful, difficult to tolerate

5 - almost unbearable

**Question 16**: How anxious/afraid were you during the examination?

1 - no anxiety/fear at all

2 - I was slightly anxious/scared

3 - I was moderately anxious/scared

4 - I was definitely anxious/scared

5 - terribly anxious/scared: couldn't wait for it to be over

**Question 17**: How long did you feel the examination was?

1 - it was not long

2 - it was of reasonable length

3 - it was long

**Question 18**: If you had to go through the examination again, how afraid would you be?

1 - I would not be afraid at all

2 - I would be slightly afraid

3 - I would be moderately afraid

4 - I would definitely be afraid

5 - I would be terrified

**Summary**

**Question 19:** If you had to undergo one of the two examinations again for your health and you were given the choice, which one would you choose?

0 - TEE

1 - MR

**Question 20:** Please briefly justify your answer to the previous question.
