# Supplementary material for: Use of a new non-contrast-enhanced BOOST cardiac MR sequence before electrical cardioversion or ablation of atrial fibrillation—a pilot study
Source: Front Cardiovasc Med. 2023 Jun 16;10:1177347. doi: 10.3389/fcvm.2023.1177347 (PMC10311645; doi:10.3389/fcvm.2023.1177347)
Supplement: Supplementary file 2 [file Table2.docx]

**Supplementary file 3**

**Results of the TEE and CMR examination distress questionnaire**

**A, TEE:**

**First, please answer the questions regarding the transesophageal echocardiography:**

**Question 1**: How informed were you about the reason(s) for the examination?

54 answers

**Question 2**: How informed were you about the examination and risks of it?

54 answers

**Question 3**: To what extent were you afraid of the investigation based on the information you had heard beforehand?

54 answers

**Question 4**: Were you given any sedative/anxiolytic medication before the transesophageal echocardiography examination?

54 answers

91%

**Question 5**: How well did the examination match what you heard in the preliminary information?

54 answers

**Question 6**: How painful was the examination?

54 answers

**Question 7**: How anxious/afraid were you during the examination?

54 answers

**Question 8**: How long did you feel the examination was?

54 answers

**Question 9**: If you had to go through the examination again, how afraid would you be?

54 answers

**B, CMR:**

**Please also answer the following questions about the cardiac MR scan:**

**Question 10**: How informed were you about the reason(s) for the examination?

54 answers

**Question 11**: How informed were you about the examination and risks of it?

54 answers

**Question 12**: To what extent were you afraid of the investigation based on the information you had heard beforehand?

54 answers

**Question 13**: Were you given any sedative/anxiolytic medication before the cardiac MRI examination?

54 answers

**Question 14**: How well did the examination match what you heard in the preliminary information?

54 answers

**Question 15**: How painful was the examination?

54 answers

**Question 16**: How anxious/afraid were you during the examination?

54 answers

**Question 17**: How long did you feel the examination was?

54 answers

**Question 18**: If you had to go through the examination again, how afraid would you be?

54 answers

**Summary**

**Question 19:** If you had to undergo one of the two examinations again for your health and you were given the choice, which one would you choose?

54 answers

**Question 20:** Please briefly justify your answer to the previous question.

- Pro-CMR justifications (**48 answers**):
  - More comfortable.
  - No physical impact.
  - No nausea, no pain.
  - No sedative.
  - Not scary at all.
  - …
- Pro-TEE justifications (**6 answers**):
  - Faster, shorter.
  - No sense of confinement.
  - Amnesia due to sedative.
  - …
